# Supplementary figures and images for: Regulation potential of transcribed simple repeated sequences in developing neurons
Source: Hum Genet. 2023 Dec 28;143(7):875–95. doi: 10.1007/s00439-023-02626-1 (PMC11294396; doi:10.1007/s00439-023-02626-1)

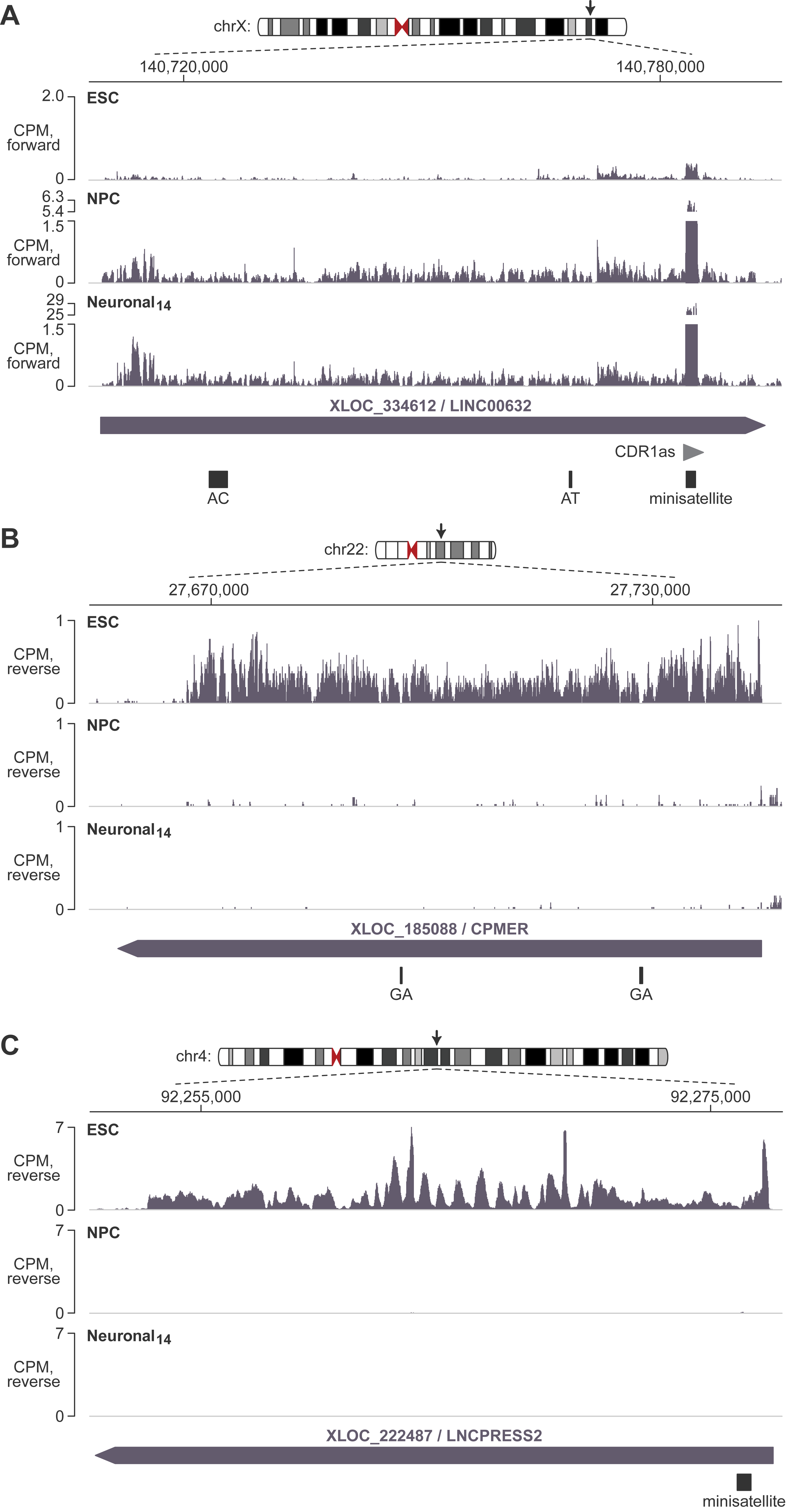

Supplement: Supplementary file 1 — Figure S1. Examples of previously characterized SRS-lncRNAs shortlisted by our workflow. Counts-per-million (CPM) normalized RNA-seq coverage plots are presented for the nuclear fraction of ESC, NPC, and Neuronal14 samples for (A) XLOC_334612/LINC00632 and CDR1as/CiRS-7, (B) XLOC_185088/CPMER, and (C) XLOC_222487/LNCPRESS2. Consistent with earlier studies (Barrett et al. 2017; Hansen et al. 2013; Jain et al. 2016; Lyu et al. 2022; Memczak et al. 2013), the SRS-lncRNAs in (A) are upregulated, while those in (B-C) are downregulated during neuronal differentiation. The arrows on the top indicate the chromosomal positions of the three SRS-lncRNA loci. The corresponding SRSs are annotated at the bottom of each panel. (TIF 1350 KB) [file 439_2023_2626_MOESM1_ESM.tif]

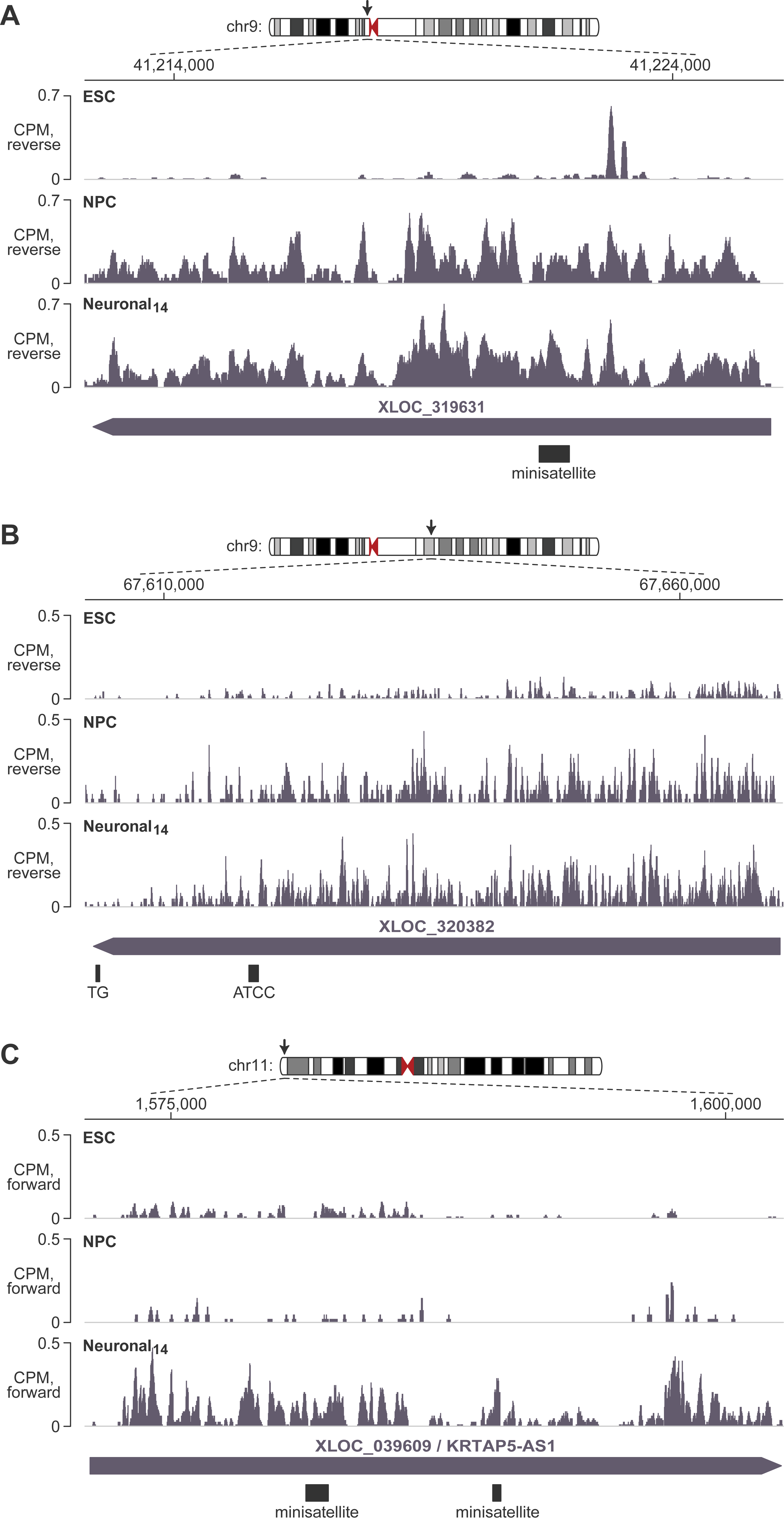

Supplement: Supplementary file 2 — Figure S2. Previously uncharacterized examples of neurally upregulated SRS-lncRNAs. (A-B) Shown are CPM-normalized RNA-seq coverage plots for the nuclear fraction of ESC, NPC, and Neuronal14 samples, illustrating the SRS-lncRNA XLOC_319631 and XLOC_320382 identified by our pipeline. (C) Similar plots for the SRS-lncRNA XLOC_039609/KRTAP5-AS1, previously proposed to function as a competing endogenous RNA in gastric cancer (Song et al. 2017), and shortlisted by our pipeline as neurally upregulated. The arrows on the top mark chromosomal positions of the three SRS-lncRNA loci. The corresponding SRSs are annotated at the bottom of each panel. (TIF 1431 KB) [file 439_2023_2626_MOESM2_ESM.tif]

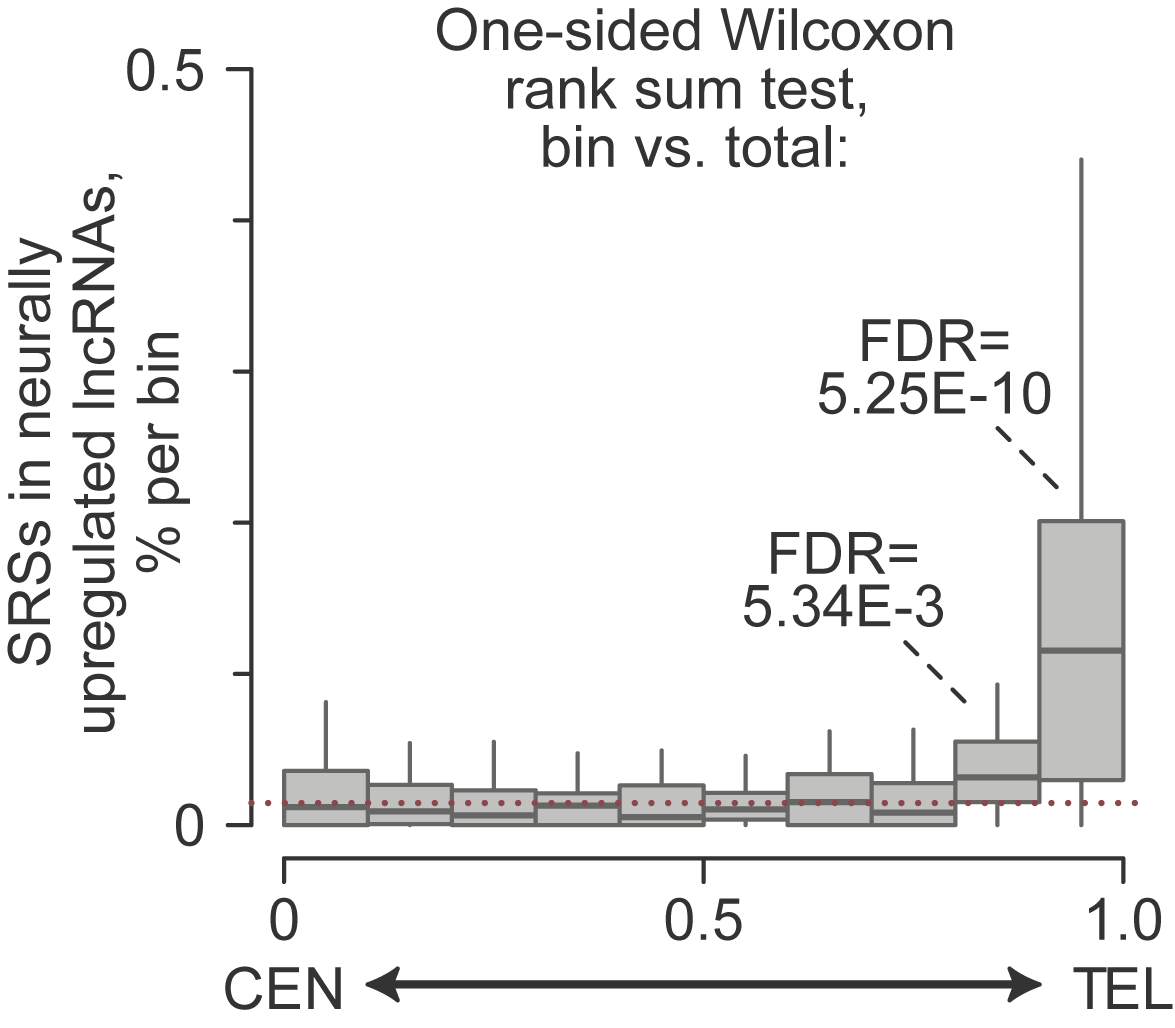

Supplement: Supplementary file 3 — Figure S3. Genomic distribution of neural SRS-lncRNAs. The box plot shows the distribution of SRSs in neurally upregulated lncRNAs along the human chromosome arms separated into 10 equally sized bins, form the middle of the centromere (position 0) to the end of the telomere (position 1). The analysis was performed similarly to Fig. 2C, except the transcripts consisting entirely of repetitious sequences were not discarded in this case. (TIF 174 KB) [file 439_2023_2626_MOESM3_ESM.tif]

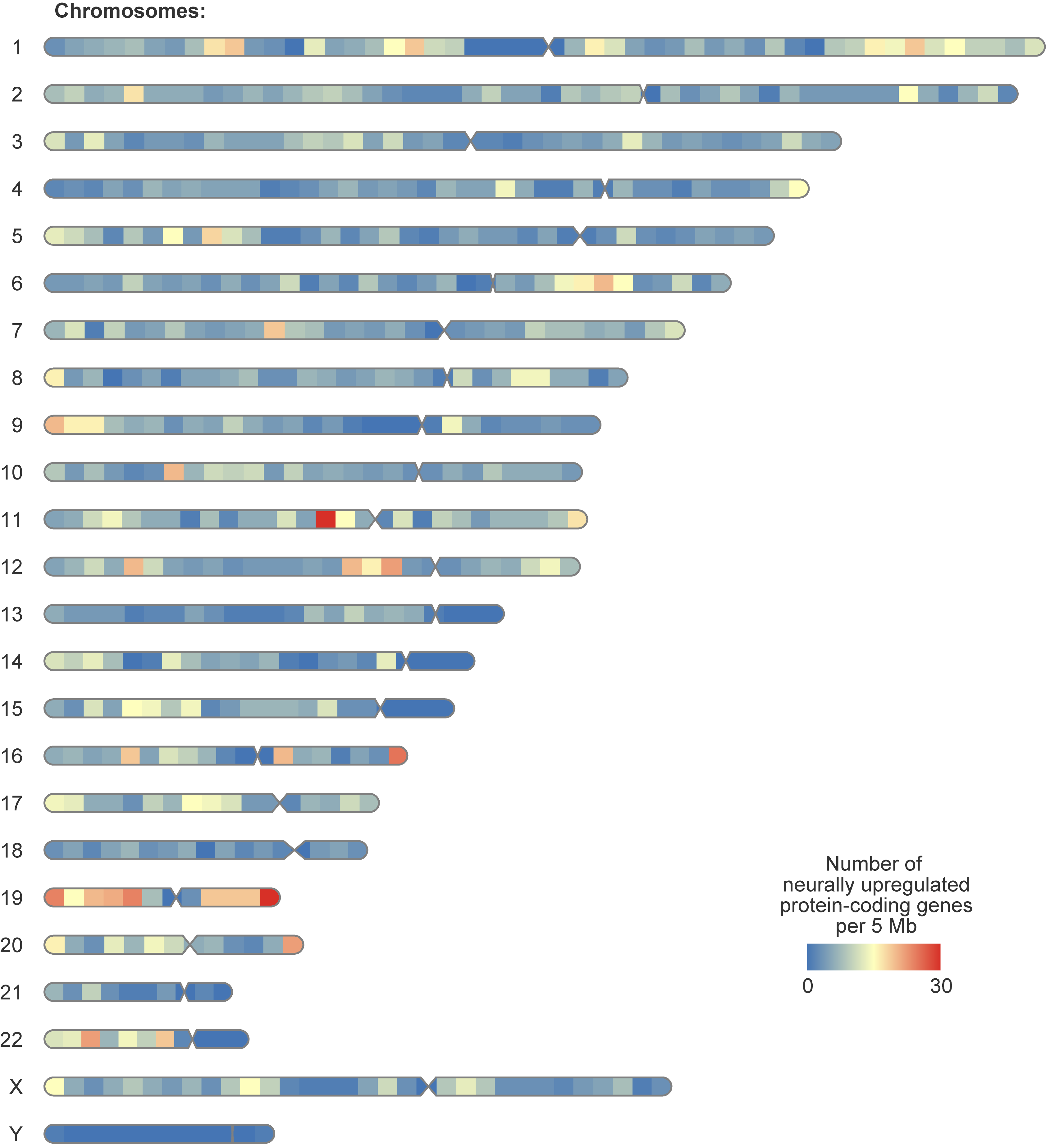

Supplement: Supplementary file 4 — Figure S4. The distribution of neurally upregulated protein-coding genes in the human genome calculated as a number of genes per 5-Mb window. Note that this pattern differs from the distribution of neural SRS-lncRNAs in Fig. 2F. (TIF 1670 KB) [file 439_2023_2626_MOESM4_ESM.tif]

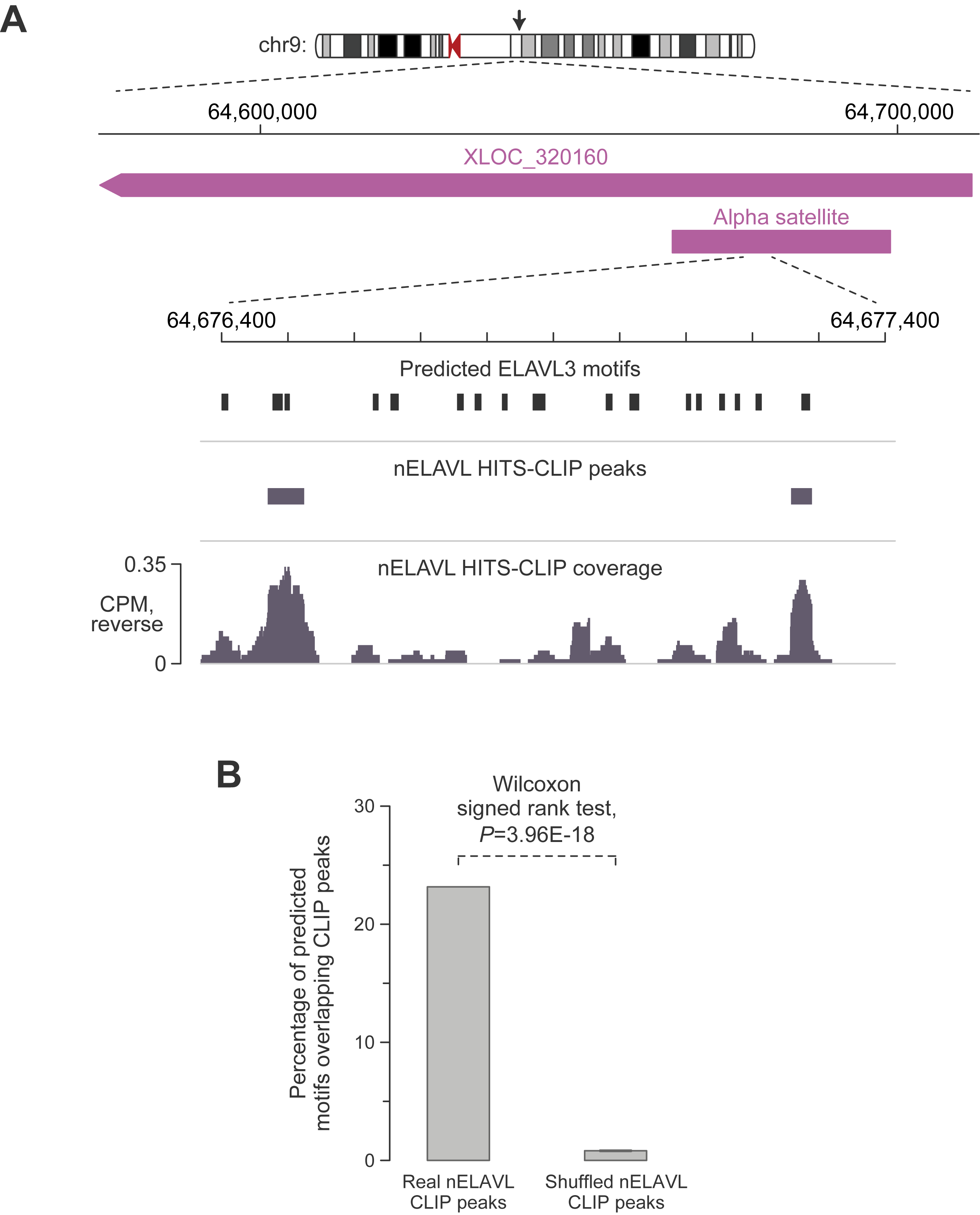

Supplement: Supplementary file 5 — Figure S5. Benchmarking RBP motif predictions using experimental data. (A) Correspondence between predicted ELAVL3 motifs and nELAVL HITS-CLIP data (Scheckel et al. 2016) for the XLOC_320160 transcript. (B) SRS-lncRNA-specific ELAVL3 motifs overlap real nELAVL CLIP peaks significantly better compared to the simulated ("scrambled") data where peak-sized windows were randomly allocated to human transcripts. The simulation was performed 100 times and the results are presented as mean±SD. (TIF 757 KB) [file 439_2023_2626_MOESM5_ESM.tif]

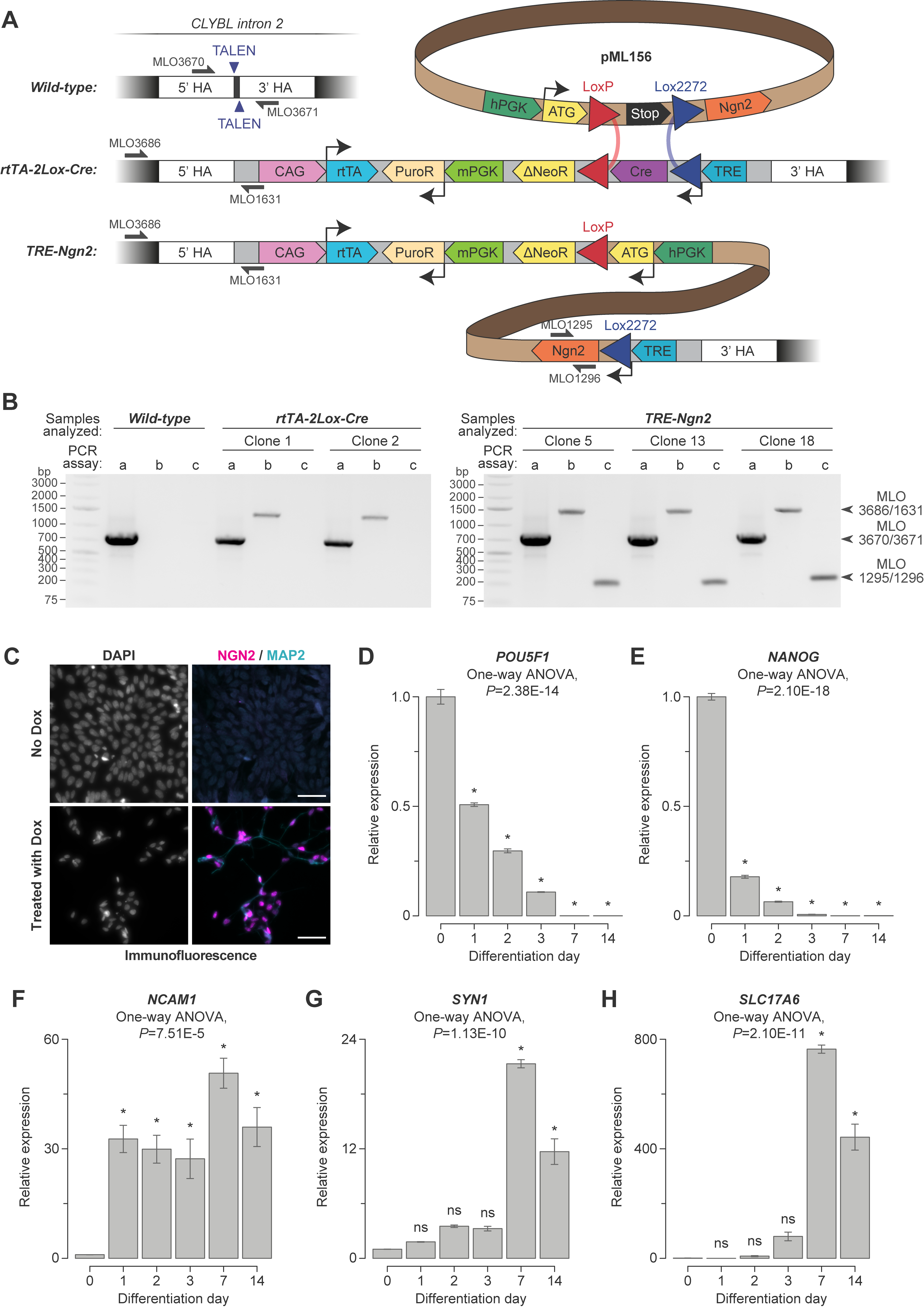

Supplement: Supplementary file 6 — Figure S6. A doxycycline-inducible system for neuronal differentiation of human iPSCs. (A) The two-step knock-in approach used in this study to generate TRE-Ngn2 iPSCs. The diagram also indicates the annealing positions of the PCR primers used to genotype the wild-type CLYBL allele (MLO3670-MLO3671, PCR assay a); the knock-in of the rtTA-2Lox-Cre cassette (MLO3686-MLO1631, PCR assay b); and the integration of the Ngn2 transgene (MLO1295-MLO1296, PCR assay c). (B) PCR genotyping results for the wild-type iPSCs and their rtTA-2Lox-Cre and TRE-Ngn2 derivatives. The corresponding amplicons are marked on the right. (C) An immunofluorescence analysis reveals the expression of the transgenic NGN2 (magenta) and the neuronal marker MAP2 (cyan) in TRE-Ngn2 iPSCs incubated with Dox for 48 hours but not in a control culture grown without Dox. DAPI was used as a nuclear stain. Scale bars, 50 μm. (D-H) RT-qPCR analyses of differentiating TRE-Ngn2 cultures for the expression of (D-E) pluripotency markers POU5F1/OCT4 and NANOG; (F) the early neuronal marker NCAM1; (G) the mature neuronal maker SYN1; and (H) the glutamatergic marker SLC17A6/VGLUT2. Data are averaged from differentiation experiments using three distinct TRE-Ngn2 iPSC clones ±SEM and compared by one-way ANOVA with Tukey’s post-hoc test. *, Tukey’s P<0.05. ns, Tukey’s P≥0.05. (TIF 5287 KB) [file 439_2023_2626_MOESM6_ESM.tif]

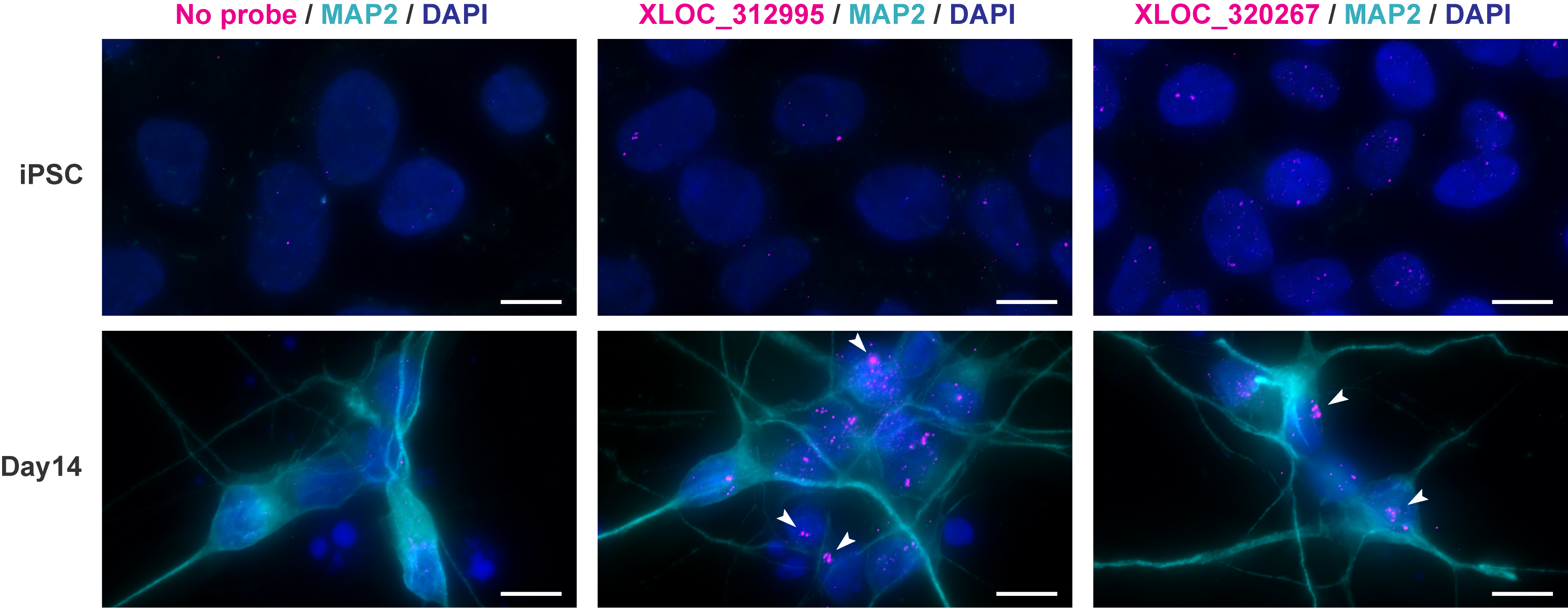

Supplement: Supplementary file 7 — Figure S7. RNA-FISH analysis of SRS-lncRNA expression. TRE-Ngn2 iPSCs and day-14 neurons were hybridized with XLOC_312995- and XLOC_320267-specific RNA-FISH probes. All samples were also stained for the neuron-specific immunofluorescence marker MAP2. Arrowheads, XLOC_312995 and XLOC_320267 form large nuclear foci in MAP2-positive neurons. Scale bars, 10 μm (TIF 7721 KB) [file 439_2023_2626_MOESM7_ESM.tif]

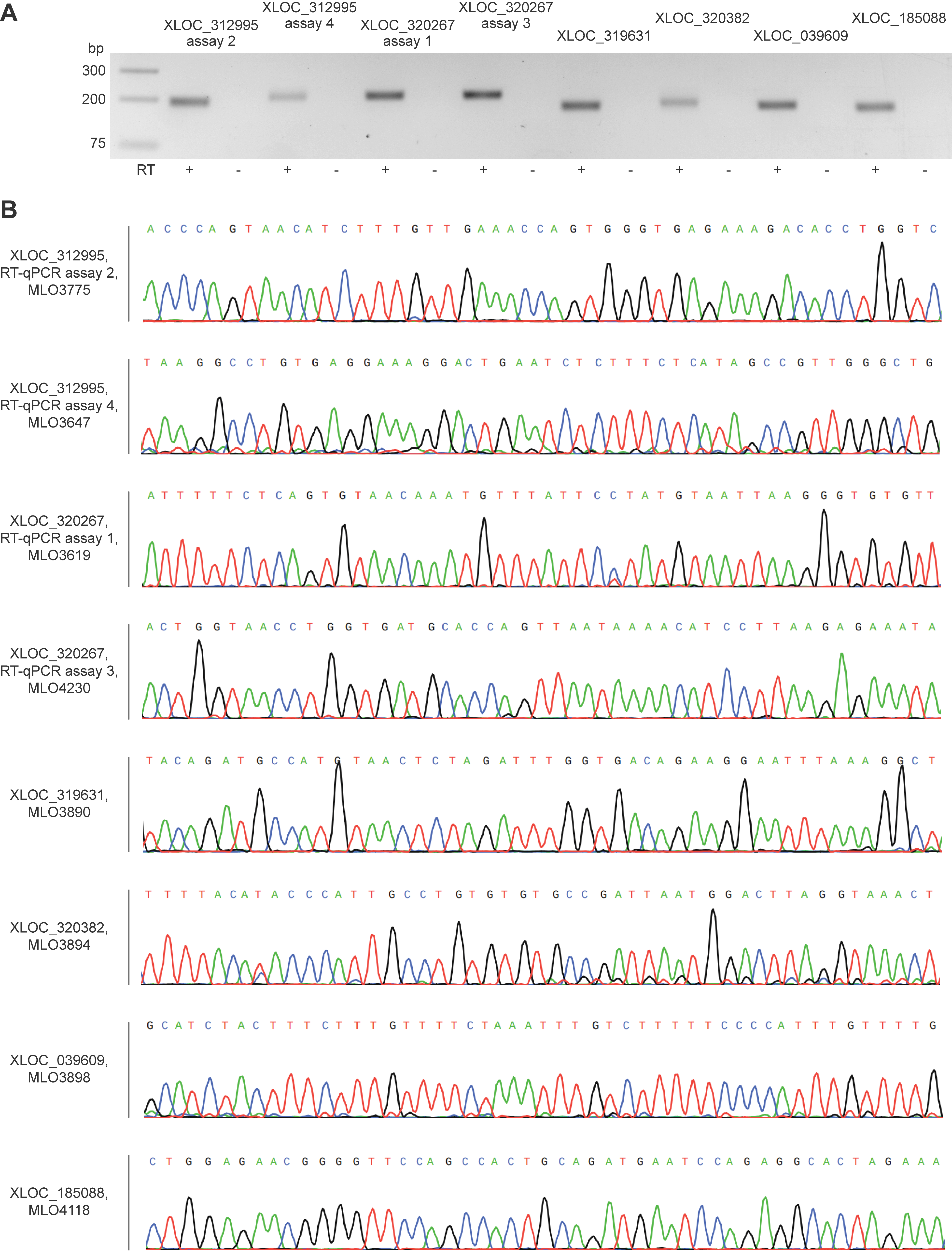

Supplement: Supplementary file 8 — Figure S8. RT-qPCR assay validation. SRS-lncRNA-specific amplification products were (A) analyzed by agarose gel electrophoresis and (B) sequenced using the Sanger protocol. (TIF 5150 KB) [file 439_2023_2626_MOESM8_ESM.tif]

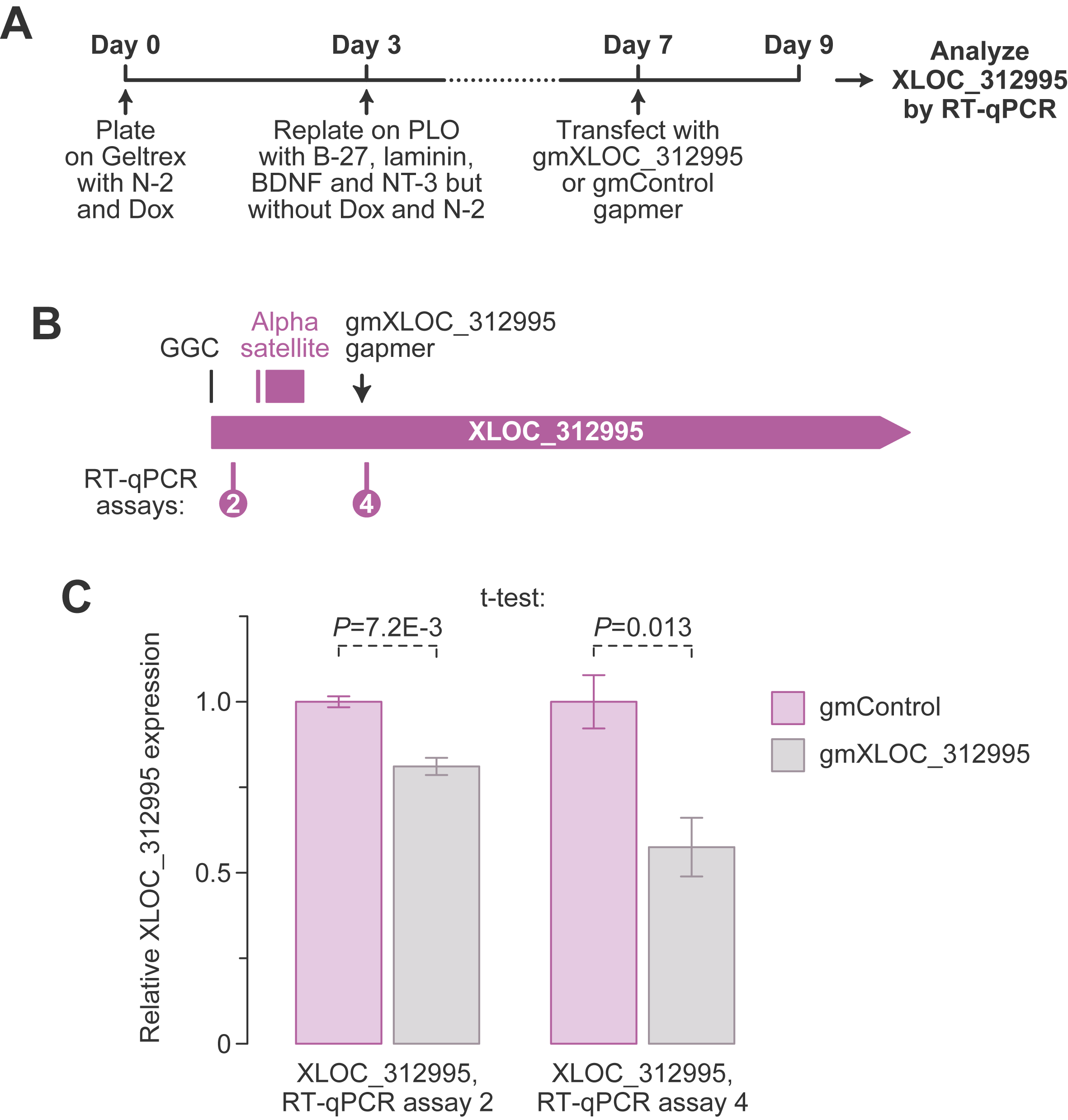

Supplement: Supplementary file 9 — Figure S9. Additional validation of the XLOC_312995 RT-qPCR assay using an antisense knockdown approach. (A) Experimental outline. (B) Annealing site of the XLOC_312995-specific gapmer gmXLOC_312995. (C) RT-qPCR assays 2 and 4 show that gmXLOC_312995 significantly reduces the abundance of the entire XLOC_312995 transcript compared to the non-targeting gmControl. (TIF 722 KB) [file 439_2023_2626_MOESM9_ESM.tif]

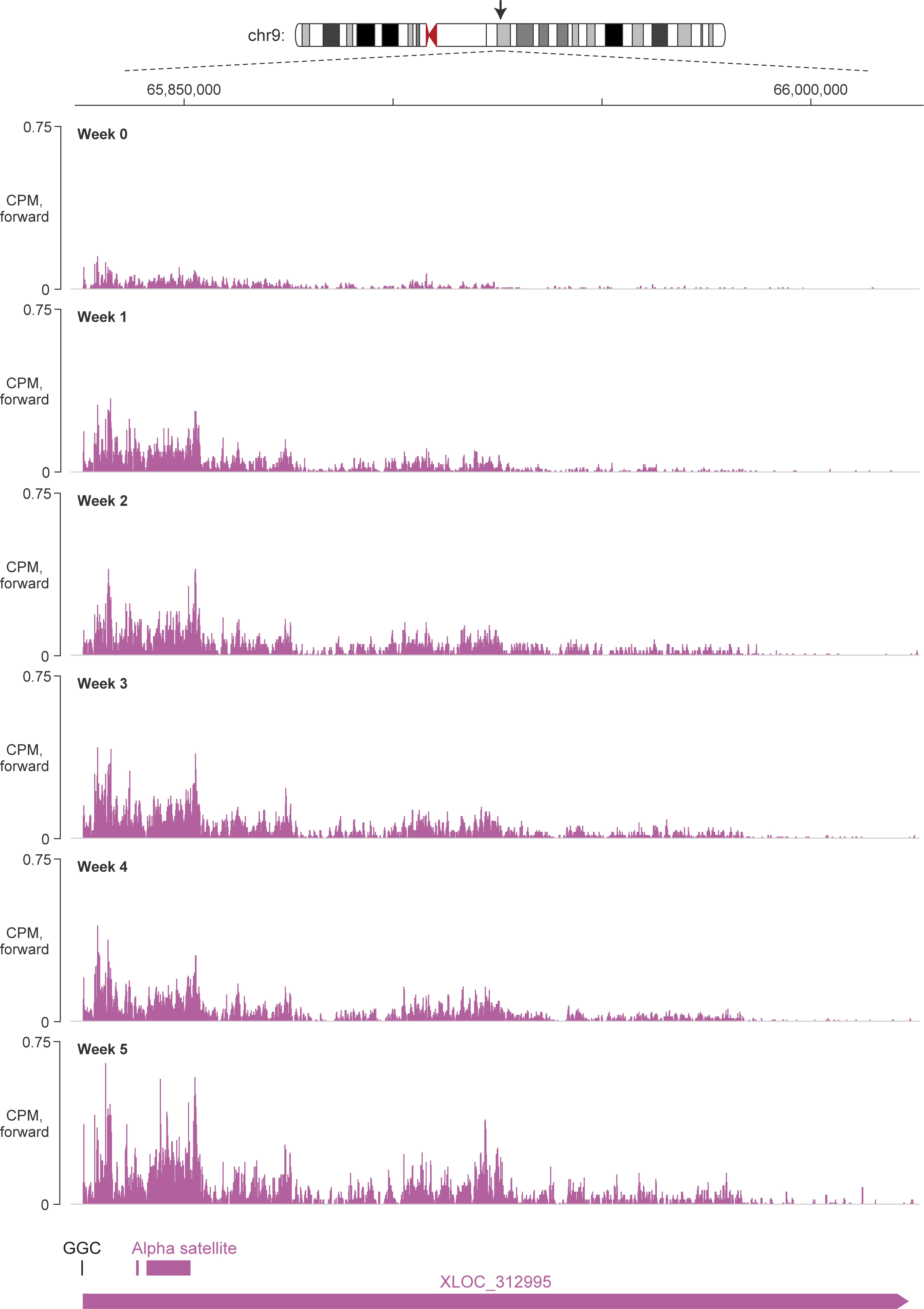

Supplement: Supplementary file 10 — Figure S10. The alpha-satellite-containing chr9 SRS-lncRNA XLOC_312995 is upregulated in developing human cortical organiods analyzed RNA-seq (Fiddes et al. 2018). (TIF 1299 KB) [file 439_2023_2626_MOESM10_ESM.tif]

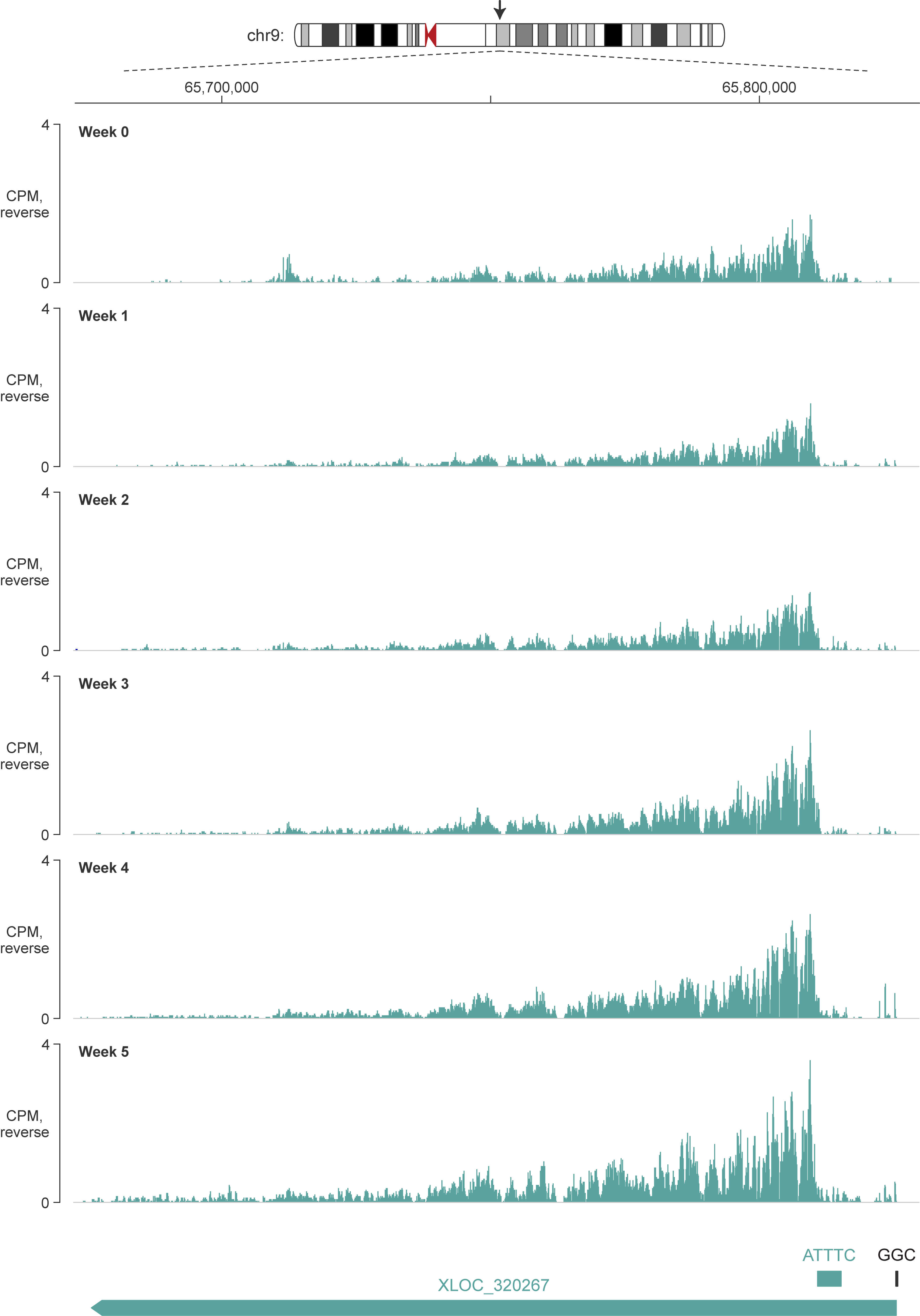

Supplement: Supplementary file 11 — Figure S11. The GAAAT repeat-containing chr9 SRS-lncRNA XLOC_320267 is upregulated in developing human cortical organiods analyzed RNA-seq (Fiddes et al. 2018). (TIF 1278 KB) [file 439_2023_2626_MOESM11_ESM.tif]

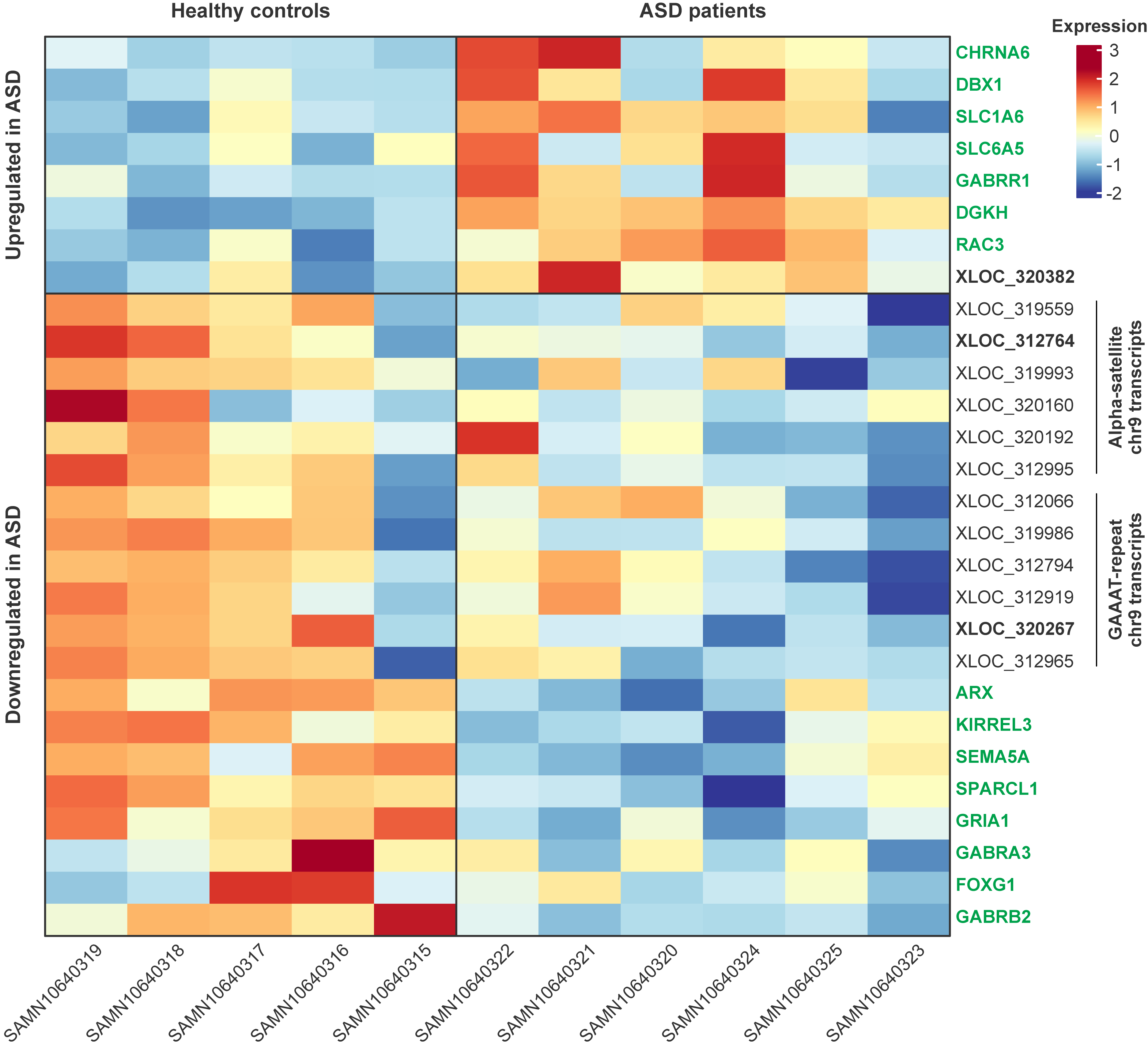

Supplement: Supplementary file 12 — Figure S12. Deregulation of SRS-lncRNA expression in ASD. The heatmap illustrates significant (DESeq2 FDR<0.05) upregulation of SRS-lncRNA XLOC_320382 and downregulation of SRS-lncRNAs XLOC_320267 and XLOC_312764 in iPSC-derived neurons from ASD patients (right) compared to healthy controls (left). Interestingly, other members of the chr9 SRS-lncRNA cluster also exhibit a tendency toward downregulation in ASD, although this effect does not reach statistical significance (the XLOC entries shown in non-bold typeset). The RNA-seq data analyzed in this panel are from (DeRosa et al. 2018). Examples of significantly regulated protein-coding genes identified by the authors are typeset in green. (TIF 3348 KB) [file 439_2023_2626_MOESM12_ESM.tif]
